# Editorial: Nontuberculous mycobacterial infections in animals and humans: pathogenesis, diagnosis, prevention, treatment, and epidemiology

**DOI:** 10.3389/fvets.2024.1532801

**Published:** 2024-12-10

**Authors:** Cinzia Marianelli, Ivo Pavlik, Giovanni Ghielmetti

**Affiliations:** ^1^Department of Food Safety, Nutrition and Veterinary Public Health, Istituto Superiore di Sanità, Rome, Italy; ^2^Faculty of Regional Development and International Studies, Mendel University in Brno, Brno, Czechia; ^3^Section of Veterinary Bacteriology, Vetsuisse Faculty, Institute for Food Safety and Hygiene, University of Zurich, Zürich, Switzerland

**Keywords:** nontuberculous mycobacteria, *Mycobacterium avium*, diagnostic tools, paratuberculosis, interference tuberculosis detection

Diseases caused by nontuberculous mycobacteria (NTM) represent a significant global public health concern. At present, NTM are defined as a group of environmental saprophytic organisms, comprising over 190 distinct species (1). Numerous NTM are opportunistic pathogens that can infect both animals and humans, resulting in a range of serious illnesses (2). Several studies have suggested that members of the *Mycobacterium avium* complex (MAC) are the most common NTM species identified in human infections and in particular in NTM lung disease (3). To date, MAC comprises 12 species, the most clinically relevant being *M. avium, M. intracellulare*, and *M. chimaera* (4). *Mycobacterium avium* is currently divided into four subspecies including *avium* (MAA), *silvaticum* (MAS), *hominissuis* (MAH), and *paratuberculosis* (MAP) based on virulence for animals and humans and other phenotypic peculiarities and specific genetic markers (4). MAH is the most clinically relevant to humans, often causing chronic pulmonary diseases and lymphadenitis in children and to pigs causing lymphadenitis in mesenteric and head (esp. submandibular) lymph nodes. MAP causes Johne's disease (paratuberculosis, PTB), a chronic granulomatous enteritis in ruminants. MAA and MAS have mostly been isolated from birds with tuberculosis-like disease.

The incidence of NTM diseases has been rising, and there is currently no established method of eliminating/eradicating the sources of infections from the environment and/or hosts (5–7). Furthermore, vaccines have yet to be developed. This Research Topic, entitled “*Nontuberculous mycobacterial infections in animals and humans: pathogenesis, diagnosis, prevention, treatment, and epidemiology*”, presents a collection of 12 studies that explore various aspects of NTM infections. Several studies address the efficacy of diagnostic tools for NTM diseases and possible interference with current *ante mortem* tuberculosis detection methods, others describe the occurrence of NTM in domestic and wild animals and the genetic diversity of NTM isolates from a wild population. Finally, a human case of NTM infection is also described.

The article by Zhang et al. reviews the applications and advancements in molecular diagnostics for identifying NTM species and subspecies. NTM infections are a growing public health concern, and accurate identification is crucial for effective treatment. Conventional methods, such as microbiological culture, are time-consuming and may not differentiate between closely related NTM subtypes. The manuscript discusses various molecular methods, emphasizing their key advantages over conventional microbiological methods, such as turnaround time, accuracy, and the ability to detect drug-resistance genes. Moreover, the article discusses the challenges in correlating *in vitro* drug susceptibility testing results with clinical outcomes. Finally, the potential of microfluidic technologies, artificial intelligence, and machine learning in enhancing the precision and efficiency of NTM identification and drug susceptibility testing, is also discussed. Stefanova et al. evaluate the efficacy of gross and microscopic investigations as diagnostic methods for MAP infection in non-vaccinated and anti-MAP vaccinated goats with subclinical infection. The article describes the prevalence of both gross PTB-compatible lesions and histopathological MAP-induced lesions in non-vaccinated goats. This finding suggests that anti-MAP vaccination may be beneficial in reducing PTB lesions and bacterial load in target organs. Furthermore, the concurrent utilization of both diagnostic tools enhances the detection of PTB lesions. Castro-Rodriguez et al. evaluate the diagnostic efficacy of two commercially available PCR-based kits for the identification and differentiation of *M. tuberculosis* complex (MTBC) and NTM human clinical isolates. The study reports a 100% sensitivity in the detection of MTBC for both kits, but a lower sensitivity for NTM identification. Gomez-Buendia et al. describe the NTM species that may act as a potential source of diagnostic interference to the intradermal tuberculin test (IDT), the most widely applied bovine tuberculosis test. The article characterizes MAH as the most abundant followed by MAA and *M. intracellulare* among MAC, and *M. nonchromogenicum* and *M. bourgelatii* as the predominant species among non-MAC members. The above four contributions highlight the limitations of the current diagnostic methods, including serological, cultural, histological, cell-mediated immunity, and molecular investigations. The combination of these methods may, however, increase the efficacy of the detection of NTM infections. Furthermore, vaccination may be a useful method of reducing the damage caused by some NTM diseases, such as PTB lesions.

Lienhard et al. examine the occurrence of NTM infection in wild animals from Switzerland, including red deer, roe deer, chamois, ibex, and badgers. The article emphasizes the opportunistic nature of numerous NTM species (particularly *M. vaccae* and MAH), which are unable to induce any macroscopic lesions in NTM-infected animals. Furthermore, the study describes the isolation of MAP from the head lymph nodes of two male red deer that exhibited no macroscopic lesions or clinical signs of disease. The study by Barandiaran et al. investigate the occurrence of NTM in four endangered Argentinian wildlife species, including giant anteater, peccary, tapir, and pampas deer. The giant anteater is identified as the species exhibiting the highest prevalence. The NTM identified include, among others, MAH and *M. intracellulare*, isolated from a range of mammalian hosts. The findings highlight the importance of NTM surveillance in conservation programs due to potential interference with tuberculosis diagnosis and public health implications. Komine et al. characterize nine *M. montefiorense* isolates from three salamander species. The strains' microbiological and genetic characteristics are analyzed, and the pathology of the infection in infected salamanders is described. The study contributes to understanding the genetic diversity and phenotypic characteristics of *M. montefiorense*, as well as the pathology of infection. In particular, phylogenetic analyses reveal that the isolates are genetically closely related, which could potentially indicate a common infection source. Turco et al. investigate the genetic diversity of *M. avium* field strains isolated from red deer in the *Stelvio* National Park in Italy. The main outcomes of this study are the genetic diversity and population structure of MAP isolates, which belong to a single major clade, indicating a clonal infection. The study also identifies two MAH isolates by investigating the same red deer population. The above four topic's articles report the occurrence of NTM and emphasize the genomic variation of NTM species in wildlife. The manuscripts contribute to our understanding of the genetic diversity and phylogenetic relationships of NTM species.

Ottardi et al. investigate the use of a commercial ELISA test in determining the seroprevalence of MAP infection in dairy herds and identifying risk factors associated with MAP spread. Because of the low seroprevalence within herds and low sensitivity of the ELISA test, the authors are unable to identify any reliable risk factors. Filippi et al. estimate the prevalence of PTB in red deer population over three culling seasons, and evaluate the relationship between the probability of being MAP-positive and individual and sampling-level variables. The large-scale serological survey by Di Marco Lo Presti et al. provides insights on MAP infection in sheep and goat herds in Sicily (Southern Italy). It reveals a high overall apparent prevalence of PTB at herd-level in sheep and goat farms, and reports animal-level prevalence data for both small ruminants. The study also indicates that the prevalence of PTB varies significantly between breeds of both sheep and goats.

While the majority of contributions to this topic focus on NTM infections in both domestic and wild animals, the study conducted by Sun et al.—which describes a rare human case of lymphadenitis caused by *M. chimaera*—underscores the fact that NTM infections represent a significant public health concern for humans as well.

Overall, these studies highlight the critical need for ongoing research and the development of comprehensive intervention strategies aimed at elucidating the mechanisms underlying NTM infections. These efforts are crucial for improving public health outcomes, enhancing veterinary management practices, and promoting effective wildlife conservation efforts, all within the context of a One Health Medicine and One Health approach.
